# Interception of an *Apis dorsata* swarm with *Tropilaelaps mercedesae* and *Kuzinia morsei* mites on a cargo vessel inbound to the United States

**DOI:** 10.3389/finsc.2026.1829350

**Published:** 2026-05-08

**Authors:** Jose Luis Ramirez, Luke R. Tembrock, Frida A. Zink, Austin Fife, Todd M. Gilligan, Yanping Chen, Jay D. Evans, Jason Mottern, Allan H. Smith-Pardo, Ron Ochoa

**Affiliations:** 1 Crop BioProtection Research Unit, USDA-ARS, National Center for Agricultural Utilization Research, Peoria, IL, United States; 2 Pest Identification Technology Laboratory, USDA-APHIS- Plant Protection and Quarantine (PPQ)- Science and Technology (S&T)., Fort Collins, CO, United States; 3 Systematic Entomology Laboratory, USDA-ARS, Beltsville Agricultural Research Center, Beltsville, MD, United States; 4 Bee Research Laboratory, USDA-ARS, Beltsville Agricultural Research Center, Beltsville, MD, United States; 5 National Identification Services, USDA-APHIS- Plant Protection and Quarantine (PPQ), Washington, WA, United States; 6 Pest Identification Technology Laboratory, USDA-APHIS- Plant Protection and Quarantine (PPQ)- Science and Technology (S&T)., Sacramento, CA, United States

**Keywords:** black queen cell virus, deformed wing virus B, giant honey bee, honey bee mites, honey bee pathogens, interception, *Tropilaelaps*

## Abstract

In 2025, a swarm of giant honey bees, *Apis dorsata dorsata*, was detected on board a cargo vessel prior to arrival at the port of Elizabeth, New Jersey, USA. *Apis dorsata* is a quarantine species not native to the United States, and further inspection of the bees uncovered 28 mites that were identified as *Tropilaelaps mercedesae* by morphological and DNA barcoding analysis. *Tropilaelaps mercedesae* is an ectoparasite of honey bees that is not yet present in the USA. An additional mite species, *Kuzinia morsei*, was also observed. Molecular screening of common honey bee pathogens in 42 intercepted bees showed a 5% prevalence of trypanosomatid infections and a 2% prevalence of American foulbrood. Additionally, Black queen cell virus (BQCV) and Deformed wing virus B (DWV-B) were detected in 38% and 7% of the samples, respectively. To our knowledge, this is the first detection of *T. mercedesae* and *K. morsei* on a vessel bound for the USA, as well as the first interception of a whole swarm of *A. dorsata dorsata*. Phylogenetic analysis of the *CO1* sequence data indicated that the intercepted bee swarm originated from a southern India lineage. Our findings indicate that exotic bee swarms can harbor parasitic mites and multiple bee pathogens, both critical to the apiculture industry. Additionally, our findings reveal that *Tropilaelaps* mites can survive extended periods associated with adult bees and in the absence of brood, suggesting greater potential for long-distance movement and introduction risk. This study provides new insights into *Tropilaelaps* dispersal and swarm-mediated pathogen movement, thereby improving our ability to predict establishment and outbreak risk crucial for countering invasive agricultural threats. The findings also emphasize that early detection, through coordinated port and shipboard surveillance, and strong interagency collaboration, is vital for reducing the risk of agricultural pest and pathogen introductions into the USA.

## Introduction

The expansion of international apicultural trade, and global trade of commodities, have facilitated the intercontinental movement of honey bee pests and pathogens worldwide (1). Among these, the ectoparasitic mites in the genus *Tropilaelaps* Delfinado & Baker 1961 are increasingly recognized as one of the most significant threats to honey bees in North America. *Tropilaelaps* spp. are native to Asia, initially found on dead honeybees and on field rats nesting near apiaries at Mataas Na Kahoy, Lipa, Philippines (2). Laigo and Morse 1968 (3) stated that the giant honey bee *Apis dorsata* Fabricius is the primary host of *Tropilaelaps clarae* Delfinado & Baker, but these mites have also successfully infested managed *Apis mellifera* Linnaeus colonies, possibly due to interactions between *A. dorsata* and *A. mellifera* (3).

Of the four described *Tropilaelaps* species, *T. mercedesae* Anderson & Morgan 2007, is considered the most widespread and destructive species to *Apis mellifera*, where it has only been found to feed on the larvae and pupae (4, 5). Infestation by this mite species results in brood mortality, malformations in the adult stage, and colony collapse. The impact on the colony is often more severe than that of the parasitic mite *Varroa destructor* Anderson & Trueman 2000, due to the shorter phoretic/quiescent phase on adult bees and faster reproductive rate of *T. mercedesae* (6, 7). *Tropilaelaps* mites not only inflict direct damage through feeding on bee larvae but can also vector honey bee viruses. *Tropilaelaps mercedesae* has been found to carry several devastating honey bee viruses, including deformed wing virus (DWV), black queen cell virus (BQCV), acute paralysis virus (ABPV) and sacbrood virus (SBV), with viral loads increasing significantly in infested colonies (8) (9, 10). Replication of deformed wing virus (DWV) has been demonstrated in *T. mercedesae* (9) and further studies have shown that mite feeding facilitates viral amplification in developing brood, with higher DWV and BQCV loads in *A. mellifera* larvae and pupae infested with *T. mercedesae* compared to mite-free controls (7). Viral transmission by *T. mercedesae* is compounded by multiple feeding wounds compromising bee larval immunity and providing favorable conditions for other opportunistic pathogens like American foulbrood (*Paenibacillus larvae*) and *Nosema* species (7) to overwhelm the infested colonies.

The dispersal mechanisms of *T. mercedesae* remain poorly understood, especially for inter-colony transmission and survival during host migration periods. Recent research by Uzunov et al. (11) demonstrated that *T. mercedesae* can successfully transfer between *A. mellifera* colonies via natural swarming events. However, their study revealed limited survival capacity, with mites surviving only 4–6 days in the absence of brood when associated with *A. mellifera*, a non-native host species (11). This short survival duration contrasts with the extended broodless periods experienced by *T. mercedesae*’ natural host, the giant honey bee (*Apis dorsata*), which spends several weeks in migratory cycles with no brood production (12, 13). This apparent mismatch between *T. mercedesae* survival capacity on non-native hosts and the bio-ecological requirements of the natural host migration patterns suggests that *T. mercedesae* may possess host-specific adaptations that allow its survival during extended broodless periods on native hosts. Understanding these survival mechanisms is critical for predicting *T. mercedesae* dispersal and developing effective management strategies.

While *T. mercedesae* is considered endemic to eastern Asia, it has been detected in South Korea, a region characterized by cold winters and a temperate climate (14). More recently this species has expanded into new regions, with new populations reported in western Russia in 2021-2022, indicating the ability of this species to establish outside the known range and overwinter in temperate climates (5). Additional detections of *T. mercedesae* were reported in Uzbekistan and Georgia in 2024 (6, 15).

Several other mite species have been observed associated with bees, among them *Kuzinia* spp (16–18). While little is known about *Kuzinia morsei* (18), several *Kuzinia* species are known to be associated with bumble bees (*Bombus* spp.) and distributed across the Neartic, Paleartic, Neotropical and Oriental biogeographic realms (17, 19). These species are known to feed on pollen, honey, bumble bee cocoon material, nematodes, and fungi, but the effect on bees living with these mites is currently unknown (16, 19). The immature *Kuzinia* deutonymphs are known to disperse on adult bumble bees (16). The co-occurrence of multiple mite species on *A. dorsata dorsata* raises important questions about potential mite-mite interactions, their impact on colony health, and whether different mites have evolved similar host-specific survival adaptations.

Herein, we report for the first time the interception of *Apis dorsata dorsata* and two mite species associated with the swarm, aboard a container vessel bound for the U.S. port of Newark-Elizabeth, New Jersey. The two mites were identified as adult *T. mercedesae* and phoretic hypopi of *Kuzinia morsei* (18). The potential economic impact of introducing *K. morsei* to the USA is unknown. We also describe pathogen screening results from the intercepted bees, since the introduction of vector-borne diseases is also a key risk factor. These findings raise serious concerns about inadvertent introductions of bees through global trade and transport.

## Materials and methods

### Vessel route reconstruction and timeline analysis

The vessel route reconstruction was conducted using documented port arrival dates, vessel characteristics, standard commercial shipping practices and availability of the exact date, time and location of the swarm interception.

**Figure 1:**
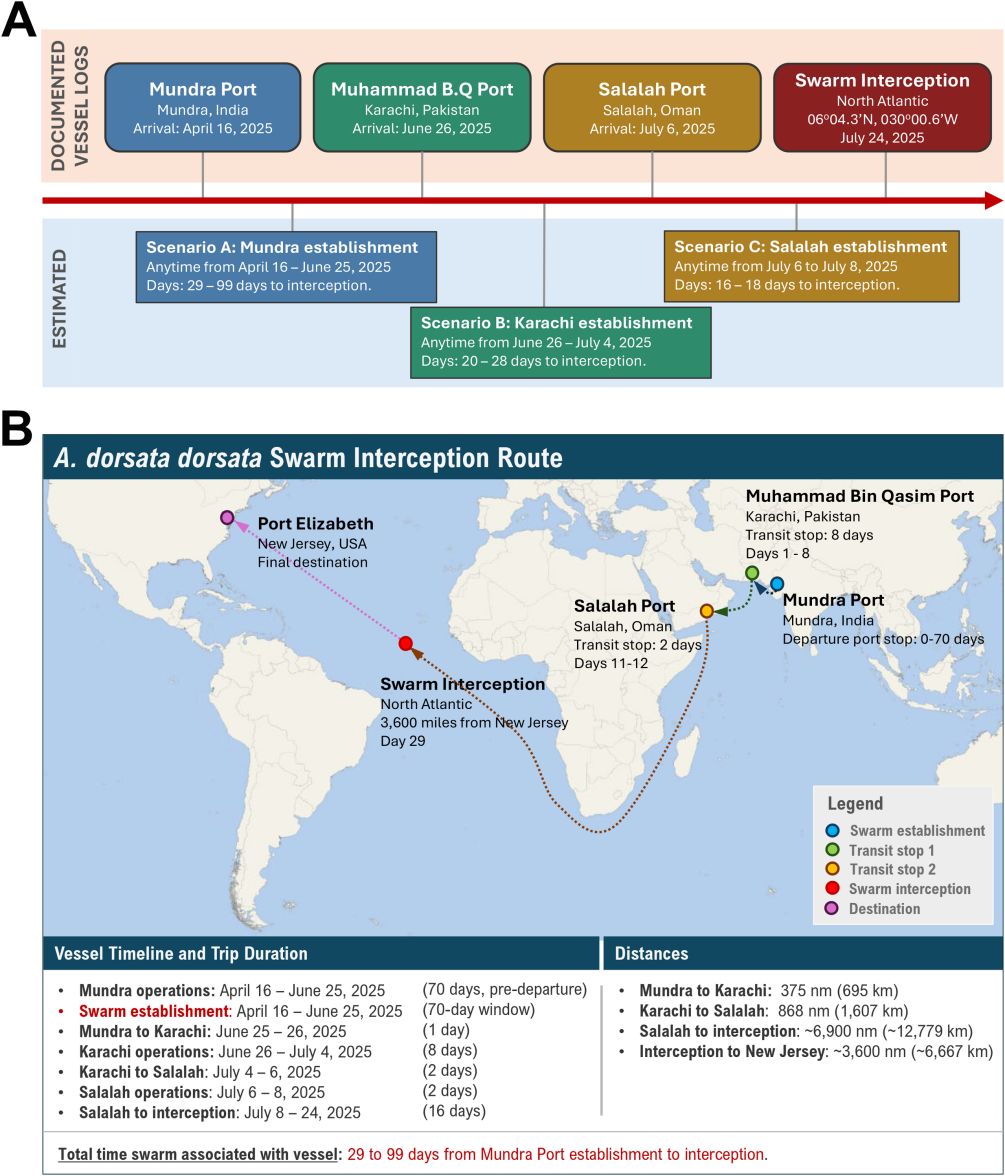
*Apis dorsata dorsata* swarm establishment and interception timeline. **(A)** Estimated establishment scenarios based on documented vessel logs and interception coordinates. **(B)** Swarm interception route from establishment to interception. The dashed lines indicate the vessel routing, and the solid circular markers show key locations.

Swarm establishment scenarios were analyzed using vessel documentation that included port arrival dates and route reconstruction. Three possible swarm establishment periods were evaluated: Scenario A: Mundra, India (April 16 – June 25, 2025); Scenario B: Karachi, Pakistan (June 26 – July 4, 2025), and Scenario C: Salalah, Oman (July 6 – 8, 2025) (**Figure 1**). Duration of swarm establishment (and mite survival) was calculated from estimated establishment to swarm interception at the coordinates 06°04.3’N, 030°00.6’W on July 24, 2025.

### Sample collection

**Figure 2:**
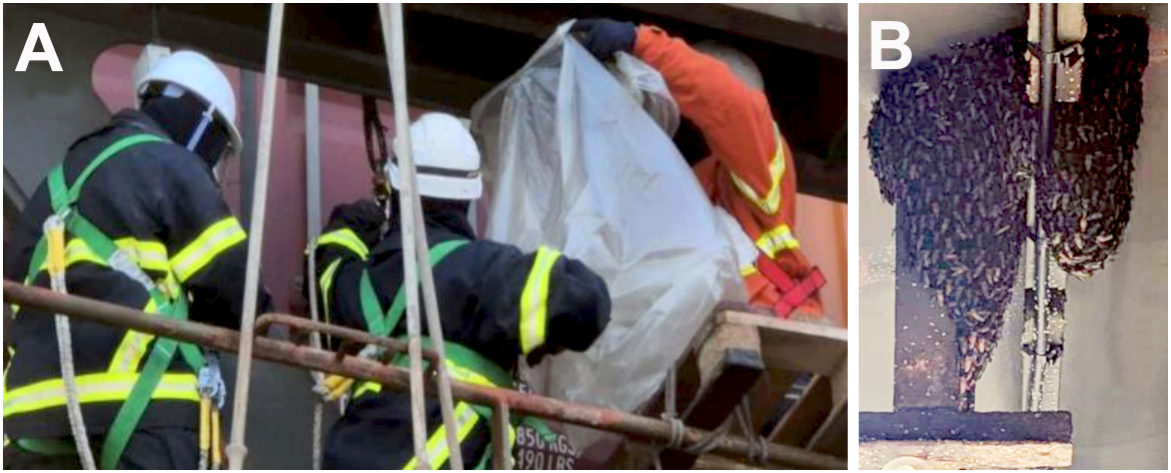
Intercepted swarm collection. **(A)** Members of the crew collecting a bivouac of bees under the guidance of the Port Director of the U.S. Coast Guard, the New Jersey Captain of the Port (COPT), and the USDA-APHIS PPQ. **(B)** View of the bivouac cluster before collection. Samples were collected while still 3,600 nautical miles from Port Elizabeth (NJ, USA).

A swarm bivouac of several hundred adult *A. dorsata dorsata* was detected by crew members of the container vessel in July 2025 (**Figures 2A, B**). Under the guidance of Customs and Border Protection and USDA-APHIS inspectors, the bees were contained by the crew in a plastic bag and frozen aboard the ship at -6 °C to prevent potential release at the port. The intercepted bees were all adults, and the swarm did not include brood or comb material. From this frozen swarm, a sample of about 70 bees was sent to the USDA-ARS’ Crop BioProtection Research Unit, at the National Center for Agricultural Utilization Research (Peoria, Illinois, USA), for initial processing and analysis. Bees were washed in 100% ethanol, and the resulting debris examined for mites using a Zeiss Stemi 508 stereoscope (Carl Zeiss Microscopy GmbH, Oberkochen, Germany). This initial inspection identified a *Tropilaelaps* mite and 10 *Kuzinia* mites. The collected *Tropilaelaps* mites and a bee leg were sent to the USDA-APHIS’ Pest Identification Technology Laboratory (PITL) (Fort Collins, Colorado, USA), for molecular confirmation. Upon this initial discovery, the whole swarm containing hundreds of bees, was requested and inspected for further presence of *Tropilaelaps* and *Kuzinia* mites at the USDA-ARS’ Systematic Entomology Laboratory (SEL) and the Beltsville Bee Research Laboratory, Beltsville Agricultural Research Center (Beltsville, Maryland, USA). The SEL’s Electron and Confocal Microscopy Unit washed bees according to the methods described in Monfreda et al., 2007 (20) and prepared samples for variable pressure scanning electron microscopy. This further inspection of the frozen swarm samples yielded an additional 27 *Tropilaelaps* and 38 *Kuzinia* mites.

### Morphological identification

Bee and mite species were confirmed morphologically using the species descriptions and taxonomic keys available in the literature (21–23), the online Lucid Keys (24) and following the *Tropilaelaps* genus description by Delfinado and Baker (2) and subsequent species-level descriptions by Anderson and Morgan (4). Variable Pressure Scanning Electron Microscopy micrography was performed using an ultra-high-resolution Schottky Field-emission Hitachi SU-7000 microscope (Hitachi Ltd, Tokyo, Japan) at the USDA-ARS Systematic Entomology Laboratory and the Electron and Confocal Microscopy Unit to support species confirmation.

### Molecular identification

From the specimens found in the swarm aboard the cargo vessel, one leg from one *A. dorsata dorsata* individual and a single whole *Tropilaelaps* specimen were sent to the USDA-APHIS Pest Identification Technology Laboratory (PITL) for molecular identification. DNA from both samples was extracted using a Lucigen MasterPure DNA extraction kit (LGC, Biosearch Technologies, Teddington, UK) following manufacturer’s instructions with modifications (25). In the case of the *A. dorsata* leg, the sample was crushed with 2.3-mm zirconia/silica beads in a 1.5-ml microcentrifuge tube by agitation on high for 1 min in a mini-beadbeater (Biospec Products, Bartlesville, OK, USA). The *Tropilaelaps* specimen was sampled non-destructively. The whole mite was placed in a 1.5-ml microcentrifuge tube and allowed to incubate overnight in tissue and cell lysis solution, after which the mite was removed and preserved for further examination while the remaining solution was processed for DNA extraction. The *CO1* DNA barcode region was amplified from both specimens using universal primers LCO1490 and HCO2198 (26). The 50.0 µl PCR mixture utilized the TaKaRa Ex Taq HS polymerase kit (Takara Bio, Shiga, Japan) and included 37.75 µl water, 5.0 µl 10X Ex Taq buffer, 4.0 µl dNTPs, 200 nM of each forward and reverse primer, 0.25 µl Taq containing 1.25U, and 1.0 µl of template DNA (except for the negative control in which water was substituted) per reaction. The PCR thermocycling conditions were an initial denaturation step of 94 °C (3 min), 32 cycles of 94 °C (20 s)/50 °C (20 s)/72 °C (30 s), and a final extension step of 72 °C (5 min), a lid temperature of 105 °C was maintained through all steps. PCR was carried out on a Bio-Rad PTC Tempo Deepwell Thermal Cycler (Bior-Rad Laboratories Inc., Hercules, CA). PCR products were visualized on 1% agarose gels to ensure correct product length and successful amplification. The PCR products were Sanger sequenced using the same primers as those for PCR amplification on a SeqStudio instrument using the BigDye Terminator v3.1 kit (ThermoFisher, Waltham, MA, USA) following the manufacturer’s instructions. Forward and reverse reads were trimmed of low-quality calls at both ends and merged into single consensus barcode sequences using Geneious Prime 2025 (www.geneious.com). The resulting barcode sequences were searched against the GenBank database using default BLASTn settings to corroborate species ID (27). Mitochondrial cytochrome c oxidase subunit 1 (*CO1*) sequences for each species from Genbank were downloaded separately and aligned using MAFFT (28, 29). Alignments were trimmed to the maximum overlap size to sample size ratio resulting in a 350bp aligned region for *A. dorsata* and a 286bp aligned region for *T. mercedesae*. *Apis florea* was used as the *A. dorsata* outgroup, and *Varroa destructor* was used for the *T. mercedesae* outgroup. Unique sequences from each species were aligned together with reference taxa. Neighbor-joining trees (30) were inferred from genetic distance matrices with 1,000 jackknife replicates, and trees were subsequently rooted using designated outgroup taxa to aid interpretation of the geographic origin of the intercepted samples.

To examine the origin of the *Tropilaelaps* mite, a haplotype network was constructed using the full CO1 sequence from the intercepted mite with the sequences used in Namin et al. (6) (indicated in **Supplementary Table S1**). The resulting 537bp alignment was used for a TCS statistical parsimony analysis (31) implemented in PopART v. 1.7 (Population Analysis with Reticulate Trees (32);).

Additional whole genome sequencing was carried out at the USDA-APHIS Pest Identification Technology Laboratory for the single *Tropilaelaps* sample using Oxford Nanopore Technology long-read sequencing. Whole genome amplification was completed to increase the total mass of DNA available for sequencing using a Qiagen Repli-g Midi kit (Qiagen, Hilden, Germany) following manufacturer’s instructions. Library preparation was completed using the ONT Ligation Sequencing Kit v14 (SQK-LSK114; Oxford Nanopore Technologies, Oxford, United Kingdom) with the NEB Next ONT Module enzymes (New England Biolabs Inc., Ipswich, MA) following manufacturer’s instructions with modifications to maximize DNA retention. The prepared library was sequenced on a GridION device for 72 hours using a MinION/GridION Flow Cell (FLO-MIN114; ONT). Sequencing data were basecalled live using the ONT Super Accuracy basecaller model. Post-sequencing, data were imported into Geneious Prime 2025. Mitochondrial and cytochrome P450 genes were pulled out using Minimap2 (33) using OR400173.1 and GCA_002081605.1 respectively as references. Successfully isolated reads were assembled using Flye v. 2.9.6 (34) set to assemble Oxford Nanopore Raw Reads with genome size varying by assembly target, and three polishing iterations.

Individual *A. d. dorsata*, *Tropilaelaps* and *Kuzinia* samples were also analyzed at the USDA-ARS Beltsville Agricultural Research Center Bee Research Laboratory, and at the APHIS-PPQ National Identification Services’ Molecular diagnostics Unit at the Smithsonian National Museum of Natural History (Washington DC, USA). Here, DNA was extracted from *A. d. dorsata* legs and whole *Tropilaelaps* and *Kuzinia* mites by placing each in 100µL of 5% Chelex-100 (Bio-Rad) in molecular-grade water. Samples were ground with a disposable pestle, and the resulting slurry was incubated at 60 °C for 10 min. The Chelex and cellular debris were pelleted by centrifugation, and the supernatant was subjected to Proteinase K digestion (0.6 mg/ml final concentration) for 60 minutes at 55°C followed by 15 minutes at 99 °C. The *CO1* barcoding region was amplified from each specimen using the primers LCO1490 and HCO2198 (26) or Lep.F1 and Lep.R1 (35) in parallel. Two microliters of each extract along with 10-fold and 100-fold serial dilutions were used in separate 25 µl PCR reactions of 94°C x 2 min, followed by 35 cycles of 94°C x 30s, 45°C x 30s, 72°C x 2 min). were carried out for each template. Prominent bands from the LCO1490 and HCO2198 pairing, based on agarose gel electrophoresis, were sequenced using Sanger sequencing. Attempts at amplification for the *Kuzinia* mite failed for both primer pairs at all dilutions. Sequence-based identifications for *Apis* and *Tropilaelaps* were concordant with results obtained by the PITL laboratory.

All *CO1* sequence data are available in Genbank under accessions (PX980322-PX980329) and long-read data is available as an SRA under BioProject (PRJNA1420927).

### Pathogen screening

Molecular pathogen screening was conducted at the USDA-ARS’ Crop BioProtection Research Unit, National Center for Agricultural Utilization Research. DNA and RNA were extracted from 42 individual bees via the ALL-prep Qiagen kit (Qiagen, Hilden, Germany) with modifications. Bee samples were macerated individually in RLT+ buffer in a microcentrifuge tube containing two metal beads using a sterile plastic mortar and further macerated in Tissue-lyser II (ThermoFisher, Waltham, MA, USA) for four rounds of 30 seconds each. Quality and concentration of DNA and RNA were assessed via NanoDrop (ThermoFisher). RNA was normalized across samples and cDNA synthesis was generated using the QuantiTec Reverse Transcription Kit (Qiagen) using 0.35 µg of total RNA. qPCR assays were conducted using a SybrGreen reagent with 1 µl of template (DNA or cDNA) and primers targeting *Vairimorpha* spp. = *Nosema* spp. (*N. ceranea* and *N. apis*), trypanosomatids (*C. mellificae*, *L. passim*, *C. expoeki*, and *C. bombi*), the bacterial pathogen American foulbrood (*Paenibacillus larvae*), and eight viruses including: Acute bee paralysis virus (ABPV), Black queen cell virus (BQCV), Chronic bee paralysis virus (CBPV), Deformed wing virus A (DWV-A), Deformed wing virus B (DWV-B), Israeli acute paralysis virus (IAPV), Lake Sinai virus (LSV), and Sacbrood virus (SBV). Further amplification was carried out by targeting bee reference genes (Actin and 28S rRNA) and microbial markers (16S rRNA and fungal ITS) to confirm nucleic acid presence and effective extraction. Primers used for pathogen screening were those from Milone and Tarpy 2021 (for targets: *Vairimorpha* spp., trypanosomatids, IAPV, DWV-A, DWV-B, ABPV, CBPV, LSV, Apis Actin, and Apis 28s rRNA) (36), Martinez et al. (for *Paenibacillus larvae*) (37), Cavigli et al. (for SBV) (38), Iredale et al. (for BQCV) (39), Nadkarni et al. (for bacterial load) (40), and Bell et al. (for fungal load) (41).

## Results

### Route reconstruction and timeline analysis

Route reconstruction revealed that the vessel arrived in Mundra, India on April 16, 2025, after which time it remained in port until June 25, 2025 at the latest. From there it transited through Karachi, Pakistan on June 26, 2025, and Salalah, Oman on July 6, 2025, and proceeded south southeast toward the Cape of Good Hope. After rounding the Cape a northwest route was plotted to cross the Atlantic Ocean toward Port Elizabeth, New Jersey. The swarm was discovered in the North Atlantic Ocean and intercepted while still 3,600 nautical miles (6,667 kilometers, at position 06°04.3’N, 030°00.6’W) from its final destination (see **Figure 1**), at which time it was reported to the US Customs officials. The cargo manifest indicated containerized shipments that included locomotive spare parts, clothing articles, hazardous materials, rice and wooden crates containing sandstone. No bulk cargo outside of a container was documented that would provide suitable habitat for the establishment or survival of a bee swarm during transit.

Swarm establishment scenario analysis indicated swarm establishment (and *Tropilaelaps* survival) of between 29 to 99 days (Scenario A: Mundra, India establishment), 20 to 28 days (Scenario B: Karachi, Pakistan establishment) and 16 to 18 days (Scenario C: Salalah, Oman establishment) (**Figure 1A**).

### Honey bee host and mite identification

**Figure 3:**
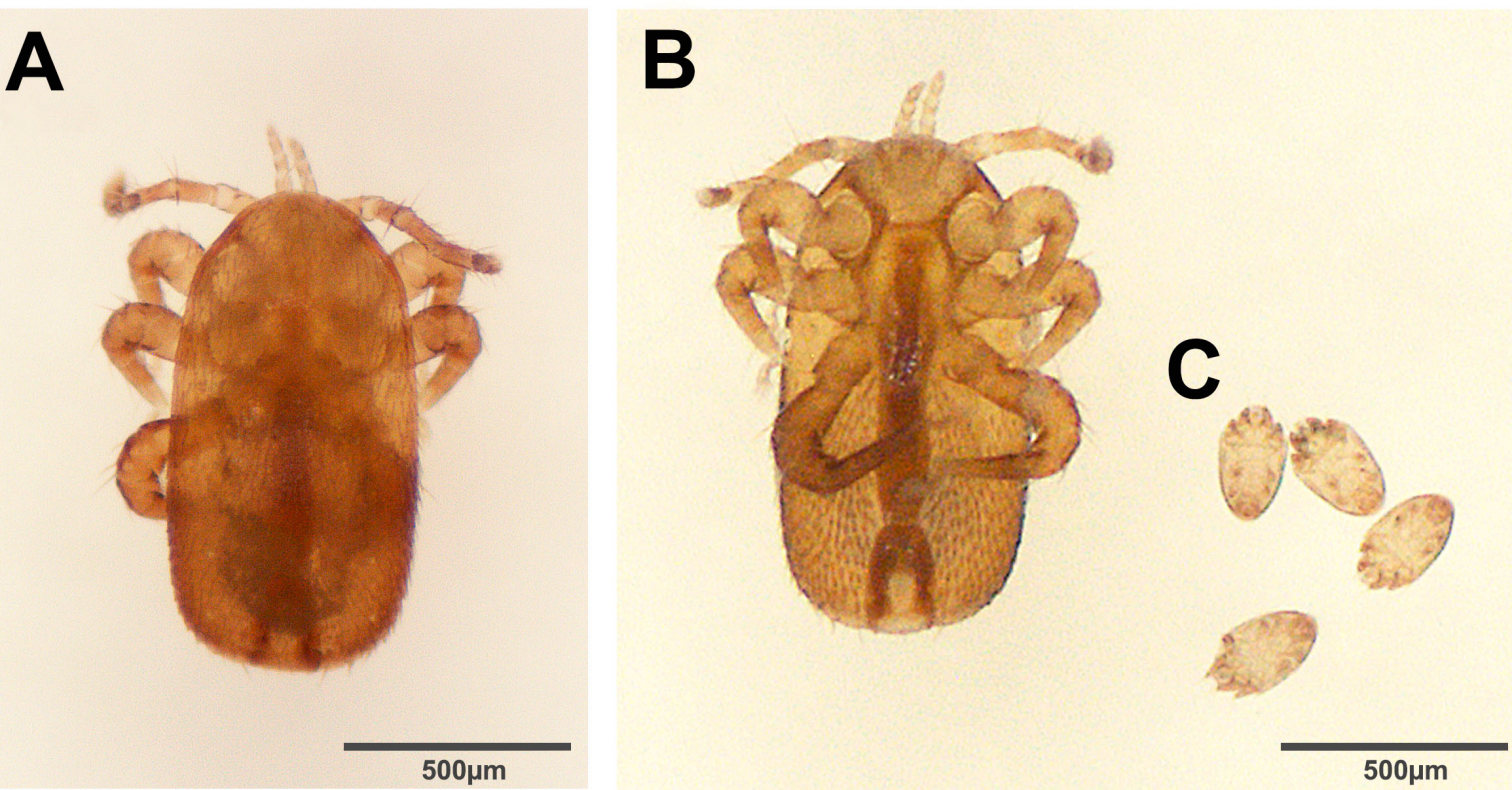
Light microphotograph of *Tropilaelaps mercedesae* from *A. d. dorsata* intercepted aboard a cargo vessel inbound for a United States port of entry. **(A)**. Dorsal view of *T. mercedesae*. **(B)** and **(C)**, Ventral views of *T. mercedesae* and *Kuzinia morsei* deutonymphs respectively, shown within the same micrograph. Panel labels are used to distinguish species, and their juxtaposition within a single image is intentional to illustrate relative body size.

**Figure 4:**
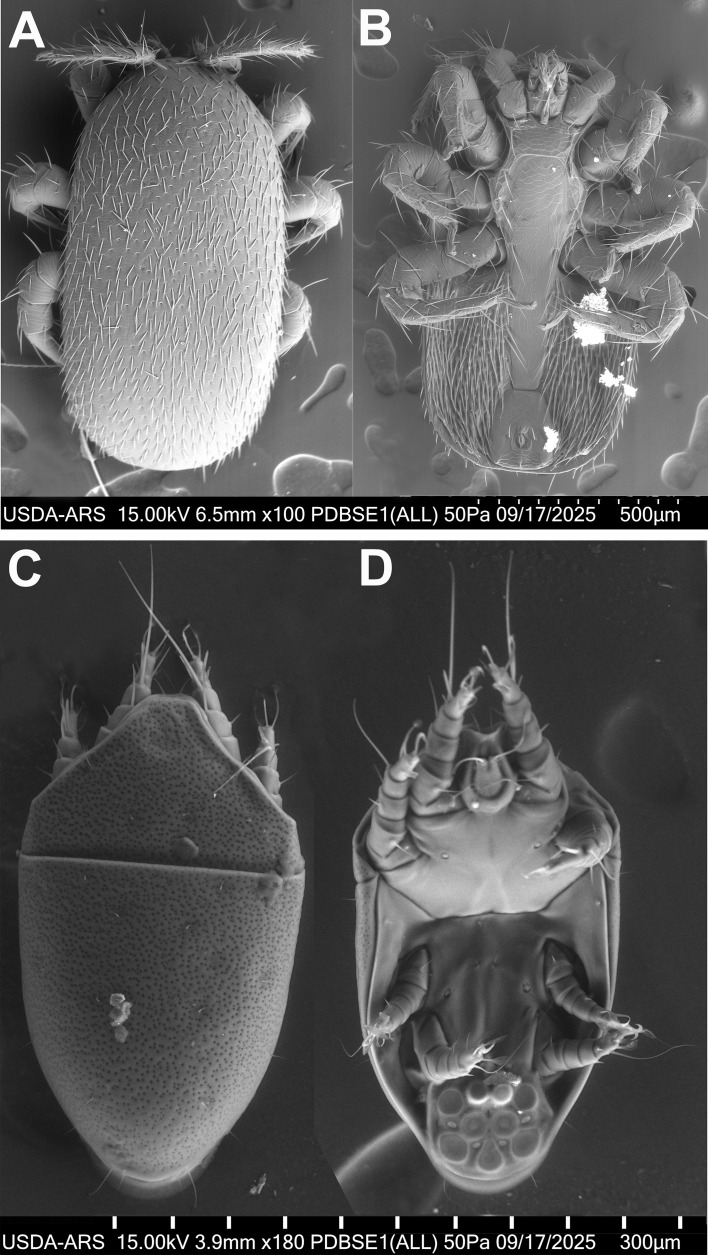
Variable pressure scanning electron microphotograph of adult female of *Tropilaelaps mercedesae* and *Kuzinia morsei* deutonymphs intercepted aboard a cargo vessel inbound for a United States port of entry. **(A)** Dorsal and **(B)** ventral view of *T. mercedesae* and **(C)** Dorsal and **(D)** ventral view of *Kuzinia morsei*. Note anal suckers on *K. morsei* ventral view **(D)** used for attachment to the phoretic host.

Bees were identified morphologically as *Apis dorsata dorsata* (**Figure 2B**) using published taxonomic keys and by comparison with museum specimens. The mite recovered from the initial bee washes was identified as *Tropilaelaps mercedesae based* on diagnostic morphology (**Figures 3A, B**), supported by electron microscopy (**Figures 4A, B**) and comparison to published descriptions (Delfinado & Baker 1961; Anderson & Morgan 2007). In addition to *Tropilaelaps*, in this initial examination, we also recovered 10 mites morphologically consistent with *Kuzinia morsei* deutonymphs based on morphological features (**Figure 3C**).

**Figure 5:**
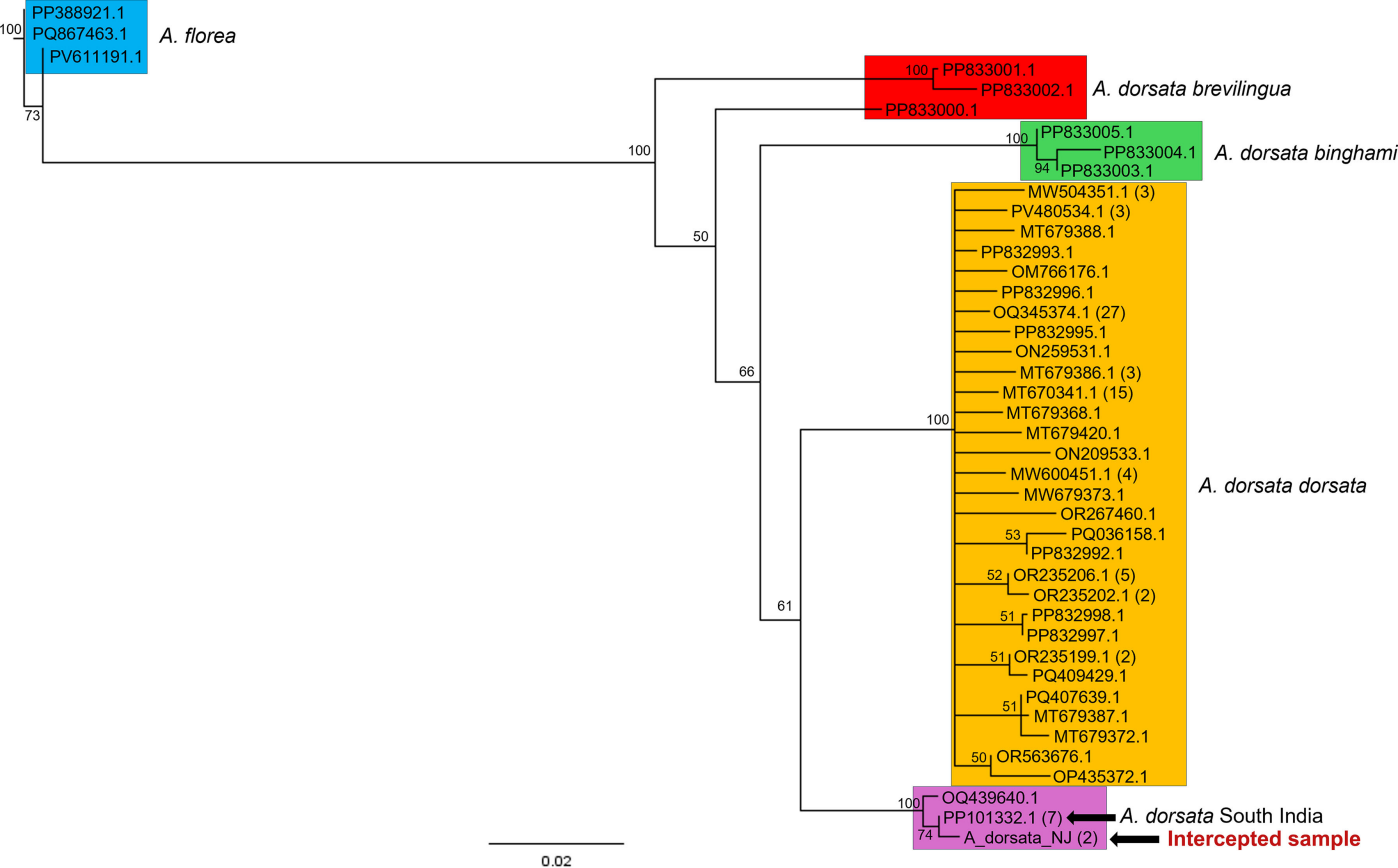
Neighbor joining trees from alignments of *CO1* DNA barcodes indicating the origin and identification of intercepted *A. d. dorsata* bees Neighbor joining tree including intercepted *A. d. dorsata* indicated by the arrow, aligned with publicly available *CO1* sequences, numbers at nodes indicate jackknife support, numbers in parentheses after GenBank accession at each terminal indicate number of GenBank accessions sharing that haplotype, colored boxes delineate *Apis dorsata* sub-lineages identified in Bhatta et al. (2024), *A. florea* sequences set as outgroup.

**Figure 6:**
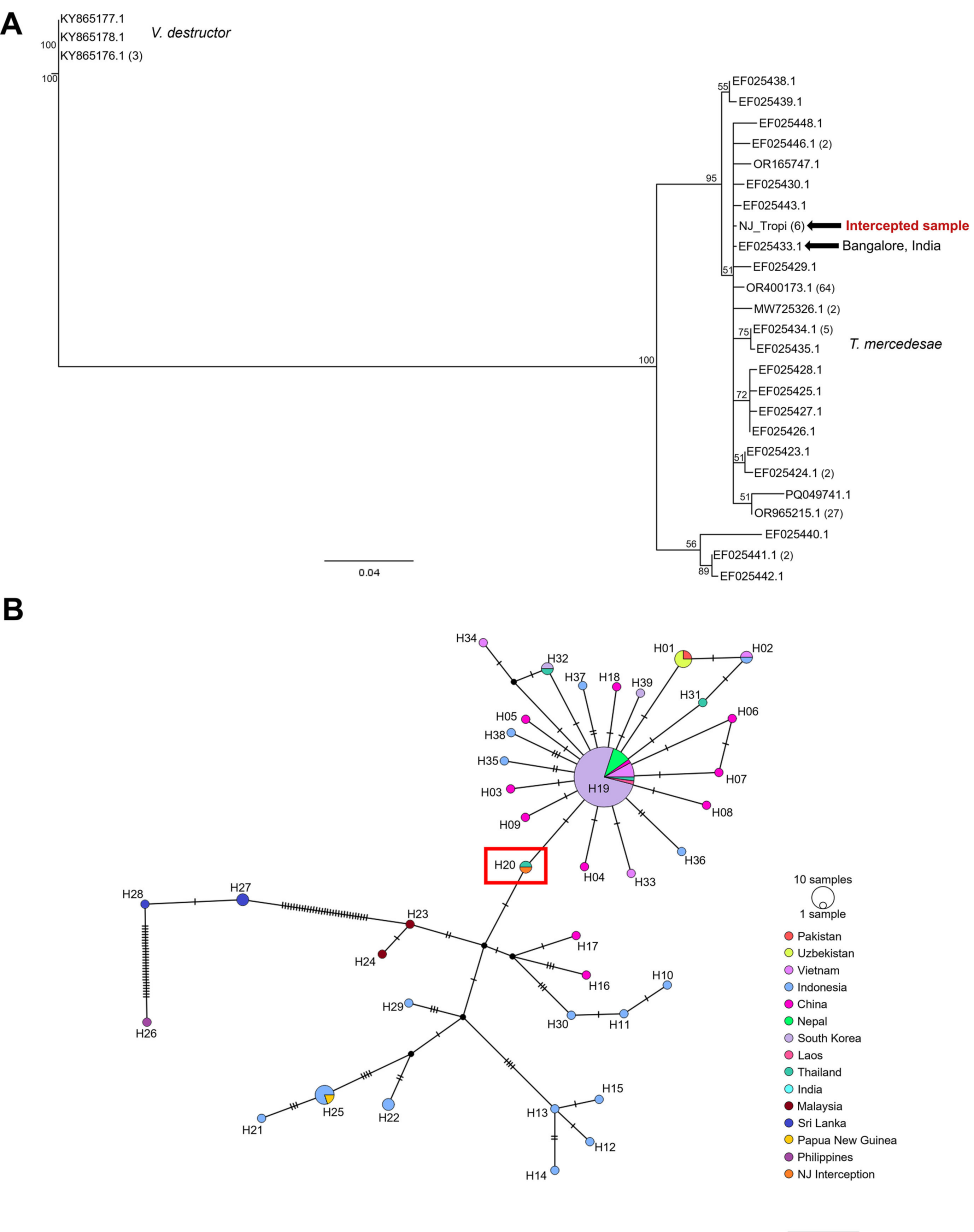
Neighbor joining trees from alignments of *CO1* DNA barcodes indicating the origin and identification of intercepted *T. mercedesae.* **(A)**. Neighbor joining tree including intercepted *T. mercedesae* indicated by the arrow, aligned with publicly available CO1 sequences, numbers at nodes indicate jackknife support, numbers in parentheses after GenBank accession at each terminal indicate number of GenBank accessions sharing that haplotype, *Varroa destructor* sequences set as outgroup. See **Supplementary Table S1** for sample details. **(B)** The *T. mercedesae* specimens collected from this interception resolve with samples from India in a TCS Network of CO1 barcodes as implemented by Namin et al., 2024. The intercepted samples are color-coded as orange in the haplotype map and resolve with H20, indicated by the red rectangle. Each circle represents a CO1 haplotype with size correlating to the number of individuals in each haplotype. Haplotypes are color-coded by country of origin; hatch marks represent single nucleotide variations between haplotypes.

Through *CO1* DNA barcoding, the intercepted bees were found to belong to a distinct clade of *A. d. dorsata* that is only known from southern India and may constitute a cryptic species or a distinct subspecies (42). The *CO1* sequence was a 100% match to a reference specimen from Karnataka, India (**Figure 5**). The *T. mercedesae CO1* sequence showed 100% sequence identity to publicly available sequences reported from multiple countries in Asia, including South Korea, India, China, Thailand, Vietnam, and Nepal (**Figure 6A**). From the long-read sequence data, a full-length assembly of *CO1* was generated as part of a full-length assembly of the mitochondrial genome. Read depth was too shallow to confidently resolve other genes of interest. The full length *CO1* assembly was used to replicate the phylogeography analysis by Namin et al. (6) and more closely resolve the possible origin of the mite. In the resulting analysis, the intercepted mite shared the same haplotype, H20 (*sensu* Namin et al. (6)), as a specimen collected from Bangalore, India (**Figure 6B**). This sequence is also highlighted in the *Tropilaelaps* neighbor-joining tree (**Figure 6A**).

Subsequent examinations of additional material at the USDA-ARS Systematic Entomology Laboratory recovered 27 additional *Tropilaelaps* and 38 *Kuzinia* mites from three sample sets. Thus, in total, the sample examinations yielded 28 *T. mercedesae* and 48 K*. morsei* mites. All *Tropilaelaps* samples were females, with no immature stages or males collected from the swarm. In the same manner, all *Kuzinia* samples were deutonymphs. These specimens were morphologically consistent with *T. mercedesae* and *K. morsei* (**Figure 4A**, B and 4C, D respectively). The *CO1* barcoding confirmed the identities of *Tropilaelaps* and *A. d. dorsata* in the corresponding samples, but DNA barcodes from the *Kuzinia* immatures could not be amplified using the standard *CO1* workflow. One additional collected mite was identified as an adult female spider mite *Tetranychus* sp. (Acariformes, Tetranychidae) with gut contents presenting a light green tone. Sample coloration and body shape across all samples (bees and mites) strongly indicate that these organisms were alive at the time of collection, with vivid body coloration and flexible, turgid bodies rather than the deflated or sunken appearance typical of post-mortem specimens.

### Pathogen screening

**Figure 7:**
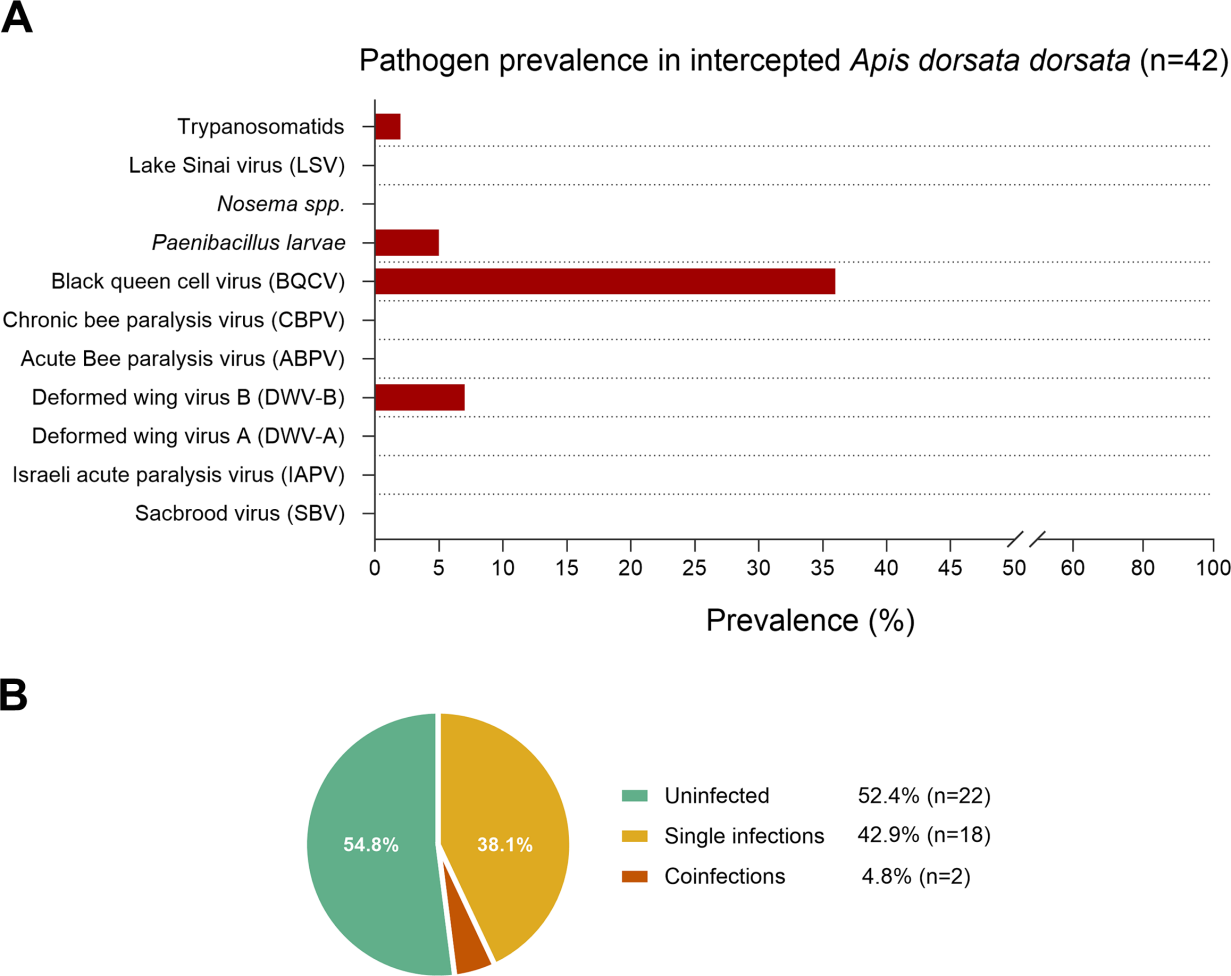
Pathogen screening of *Apis dorsata dorsata* intercepted aboard a cargo vessel inbound for a United States port of entry. **(A)** Prevalence of detected pathogens via qPCR is shown as percentage of positive individuals in the analyzed sample set (n=42). Detected pathogens included trypanosomatids (5%), *Paenibacillus larvae* (2%), Black queen cell virus (38%) and Deformed wing virus (7%). No other screened pathogens (*Nosema* spp., ABPV, CBPV, DWV-A, IAPV, LSV, SBV) were detected. **(B)** Proportion of giant honey bees with no detected infection, single pathogen infection, or coinfection by more than one pathogen.

Our pathogen screening of *A. d. dorsata* did not detect any *Nosema* spp. but detected two individuals with trypanosomatid infection. The bacterial pathogen *Paenibacillus larvae* (causative agent of American Foulbrood) was detected in 2% of the bees evaluated. BQCV was detected in 38% of individuals, while DWV-B was found in 7% of the samples. All other RNA viruses tested were negative (**Figure 7A**). From the samples examined, 52.4% were uninfected (n=23), 42.9% (n=18) had a single infection, while 4.8% (n=2) were coinfections (**Figure 7B**), with one sample presenting trypanosomatid and BQCV infections, and the other coinfected with BQCV and DWV-b viruses. Reference genes amplified consistently at qPCR cycle threshold (Ct) Ct=10 (*Apis* 28s rRNA) and Ct=27 (actin) (36), while pathogens showed higher Ct values (>Ct=30). Given that the intercepted samples have not been maintained under optimal storage conditions for RNA preservation, and that suboptimal sample storage significantly affects viral detection sensitivity in bee samples (43), the prevalence estimates should be considered as lower-bound estimates. The higher pathogen Ct values may reflect low viral loads, RNA degradation, or both, potentially leading to underestimation of true pathogen prevalence. All pathogens detected during our molecular screening are already present in the United States and are not considered quarantine pathogens; however, they are of significant concern for honey bee health, as several (i.e. *Paenibacillus larvae* (American foulbrood) and Deformed wing virus) are strongly associated with colony morbidity and collapse.

## Discussion

To our knowledge, this interception represents the first detection of *Apis d. dorsata* as well as *Tropilaelaps mercedesae* and *Kuzinia morsei* on cargo inbound to the USA. Morphological and molecular evaluation from multiple laboratories corroborated that the *Tropilaelaps* specimen is consistent with descriptions and DNA barcodes for *Tropilaelaps mercedesae* (2, 3). The *A. d. dorsata* specimens were identified as such by comparing their *CO1* sequence with published barcodes and were morphologically congruent with published descriptions and museum specimens of *A. d. dorsata.* The *A. d. dorsata* subspecies was resolved in a distinct clade (with 100% JK support) within *A. d. dorsata;* found only in southern India and morphologically indistinguishable from *A. d. dorsata* ( (42); **Figure 5**). These results place the intercepted swarm within a southern India lineage, based on the resolution of these individuals in the NJ tree. This is relevant because southern India is a region where *Tropilaelaps mercedesae* has been reported, and it provides a plausible source for the mites recovered from the swarm. Our phylogenetic analysis conducted on the intercepted *Tropilaelaps* mites, places it within a well-supported clade, also, alongside sequences of Indian origin, including a reference from Bangalore. The haplotype network analysis (albeit limited by available sequences) corroborated this placement, with the intercepted sample (H20) falling within an India-associated haplotype, further supporting an Indian geographic origin for this interception. The finding is particularly significant given the history of invasions of *Tropilaelaps* into new regions, which have repeatedly followed host movements into new areas. The successful introduction of *T. mercedesae* is predicted to be a major threat to U. S. apiculture because it has become a serious pest of honey bees both where it is endemic and where it is introduced (5, 6).

The threat posed to honey bees by *Tropilaelaps* is substantial and derives from their distinctive biology and epidemiology. These mites reproduce faster than *Varroa destructor* due to their short lifecycle and ability to feed on both pre- and post-capped brood (7, 44–46). Experimental studies have shown that infestations of *Tropilaelaps* lead to multiple feeding wounds per larva or pupa, resulting from repeated penetration of the bee cuticle by the mite’s sharp chelicerae (7, 47). This extensive tissue damage leads to weight loss, deformities in the adult bee, and increased mortality (7). In addition to the direct feeding damage that *Tropilaelaps* mites inflict on bees, they are also competent vectors of DWV, with active replication confirmed within the mites (9, 48). Dissemination of the virus through feeding facilitates viral proliferation in brood, with pupae infested with *Tropilaelaps* exhibiting higher DWV and BQCV loads compared to *Tropilaelaps*-free controls (7).

Our pathogen screen of intercepted bees found no microsporidia but revealed BQCV infection in about a third of the bees tested. This aligns with the virus’ association with mite feeding wounds (7). Our pathogen survey analysis must be interpreted within the context of sample storage limitations. The prevalence estimates we observed (38% BQCV, 7% DWV-B) likely reflect the suboptimal sample preservation following the interception and during transport to the lab (at -6°C rather than at -80°C). Recent studies have demonstrated that storage conditions can significantly impact viral RNA detection in bee samples (43). Despite these limitations, our findings add to the limited research on pathogen associations with *A. d. dorsata* bees. The detection of multiple pathogens, even under suboptimal preservation conditions, demonstrates that multiple regulated bee pathogens circulate in wild giant honey bee populations.

Sample coloration, turgidity and body positioning strongly indicates that all collected organisms (bees and mites) were alive at the time of collection. The presence of *Tropilaelaps* mites in a broodless *A. d. dorsata* swarm that stayed aboard a ship for several weeks at sea is biologically and epidemiologically significant, demonstrating that adult *Tropilaelaps* mites can survive extended periods associated with adult bees and without access to brood. Although *Tropilaelaps* mites are considered to be highly dependent on brood, and do not exhibit prolonged phoretic behavior compared to *Varroa destructor*, previous studies have shown that they can remain alive on adult bees for up to six days in conditions devoid of brood (49–52).

Route reconstruction and vessel timeline analysis revealed three potential establishment scenarios where the *A. d. dorsata* swarm might have established on the vessel. Thus, the period of association of the broodless swarm (along with *Tropilaelaps and Kuzinia* mites) with the vessel varies significantly depending on the establishment timing and location, ranging from 29 to 99 days (Scenario A: Mundra, India), 20 to 28 days (Scenario B: Karachi, Pakistan) and 16 to 18 days (Scenario C: Salalah, Oman). The molecular analysis of both the *A. d. dorsata* bees and *Tropilaelaps* mites confirmed an Indian subcontinent geographic origin for the swarm, which combined with the documented extended vessel operations at Mundra Port (70 days), provides strong support for swarm establishment at Mundra (India). The scenario B (Karachi, Pakistan) swarm establishment is the most conservative and remains biologically plausible (based on geography and length of time on the vessel). However, this scenario lacks the molecular support for Pakistani geographic origin, and it has a shorter transit stop compared to the longer Mundra operations period; which provides an ample opportunity to establish aboard the vessel with possible access to land-based resources to forage and only adds, at a minimum, one day to the survival window over the Karachi scenario. The third scenario, establishment at the Salalah Port (Oman, Scenario C), is highly unlikely, given that there are no published records of *A. d. dorsata* in Oman (53), and it could only occur if the swarm moved from a different vessel during harbor operations at this location.

Although a Mundra establishment appears to be extreme for a broodless swarm to survive, Robinson (12, 13) found that the time bivouacs (migrating swarms) of *A. d. dorsata* stayed at a particular location, ranged widely from two hours to more than 57 days on their migration route. The remarkable capabilities for migration of this species, which include seasonal migrations of up to 200 km and the ability to fly 1.9 km to forage, coupled with its adaptation to build open nests suspended from elevated structures and survive exposure to sun, wind and rain (12, 13, 54, 55), make this scenario entirely plausible.

The observation that *Tropilaelaps* mites from this interception were surviving at least 29 days without access to brood when associated with its natural host *A. d. dorsata*, is a significantly longer survival duration compared to the 6-day maximum reported for *A. mellifera* by Uzunov et al. (11). This extended survival period aligns well with *A. d. dorsata*’s migratory ecology, in which seasonal migrations last several weeks during which colonies experience prolonged broodless phases (12, 13, 54). These data are important because it demonstrates the resilience of migrating *A. d. dorsata* swarms when there are no adequate conditions for a permanent nest (such as during oceanic crossings), and their potential capacity for foraging for resources when the vessel was stationed at ports (India, Pakistan and Oman), enabling survival for extended periods without stored resources in the absence of comb. This survival capacity also suggests the existence of host-specific adaptations by *Tropilaelaps*, that may have co-evolved with the natural migratory behavior of *A. d. dorsata*. These potential adaptations would likely include physiological tolerance to starvation and stress, modified host-seeking behavior, or exploitation of alternative nutritional sources available on the natural host but absent in *A. mellifera* (56). Intact swarms might also provide dense adult-host contact that facilitates mite survival during broodless swarming periods, thus serving as a viable transport for *Tropilaelaps* mites, and increasing the potential for establishment if brood hosts were to become available soon after arrival (8, 47).

Thus, the extended survival capacity on native hosts has significant implications for understanding the invasion and dispersal potential of *T. mercedesae*. In contrast to the limited dispersal, due to short survival periods on *A. mellifera* (11), the 3–4 week survival on its native host *A. d. dorsata* would facilitate long-distance dispersal during migration cycles. This could explain the historical distribution patterns of *T. mercedesae* across Southeast Asia (4, 57). These findings would also suggest that successful *T. mercedesae* establishment is dependent not only on suitable climatic conditions but also on associations with the specific host (47).

Little is known about *Kuzinia morsei*, but they were encountered in larger numbers than *Tropilaelaps*, with more than 40 individuals from alcohol washes of the intercepted *A. d. dorsata*. A *Kuzinia* mite was found still attached near the rastellum and another mite located on the thorax of *A. d. dorsata*. Our records show that this is the first documented interception of *K. morsei* in a vessel bound for the United States. The original description of *Kuzinia morsei* is from samples collected by Roger Morsei in the Philippines in 1968, and its association with *A. d. dorsata* was not recognized until its description 22 years later in 1990 (18). It took another 26 years, from the presently described interception, for this association to be corroborated. Given that the likely origin of the intercepted materials is India, we predict that these mites could occur in other parts of Asia. The economic impact of *Kuzinia* species is unknown, and they could not be identified with *CO1* DNA barcodes as PCR failed to produce amplicons from these samples. It should be noted that *Kuzinia morsei* is currently under taxonomic revision and the resulting resolution may help to better understand the importance of this species. The presence of a *Tetranychus* spider mite among the samples is likely accidental but given that the material gut contents were still green, it is indicative of some recent contact with plant material by the intercepted bees. The extended survival we observed in *T. mercedesae* may not be unique among other *A. d. dorsata* ectoparasites, as *Kuzinia morsei* likely faces similar selective pressures during migration periods (4) but currently little is known about its biology or ecological role.

*Apis dorsata dorsata* is typically associated with forested habitats, where colonies nest openly on tall trees or cliffs in tropical and subtropical regions of South and Southeast Asia (12, 58, 59). Our observation and previous reports indicate that swarming or absconding by *A. d. dorsata* can include highly modified or urban-adjacent environments (60, 61). From a biosecurity perspective, the ability of *A. d. dorsata* to occupy modified landscapes and to move over long distances during foraging or migration cycles, may increase the likelihood of contact with human-mediated transport pathways, including vessels and associated port environs.

The results found here support previous remarks by Smith-Pardo et al. (62) about the importance of vigilance at ports of entry to prevent the introduction of non-native invasive species of honey bees (genus *Apis*) and their parasites and potential diseases into the USA. Social insects are particularly serious because they can survive for long periods of time in adverse conditions and because a single queen or swarm can found a colonial population in a short period of time. In the case of giant honey bees, this is particularly problematic as they are close relatives to the only species present in the USA, *Apis mellifera* (also introduced but naturalized and exploited commercially) and can compete with this species or serve as hosts of parasites that can easily jump from one species to another. In addition, recent reports of *T. mercedesae* establishment in Europe (5), further highlight the species ability to adapt to non-native habitats.

The interception described here resulted in the immediate destruction of the foreign swarm, preventing the potential establishment of *Tropilaelaps* in the USA and demonstrating that invasive bee pests can arrive as hitchhikers with cargo. Collectively, this interception underscores how exotic species of honey bees can serve as vehicles for the transport of parasitic mites and associated pathogens that threaten domestic beekeeping and agriculture. These findings suggest that stowaway pests may be transported on inbound vessels regardless of declared cargo type, and that coordinated port and shipboard surveillance, together with communication with shipping companies, can strengthen early detection and risk awareness.

## Conclusions

Molecular phylogenetic analysis of the intercepted bees and mites confirmed their placement within a distinct clade of *A. d. dorsata* and *Tropilaelaps* mite endemic to India, establishing the port in Mundra (India) as the most probable origin of the swarm. Combined with the vessel’s travel timeline and official communications of the swarm interception, we estimate that the broodless swarm and mites survived aboard the ship between 29 to 99 days from establishment to interception, with 16 of those days at sea with no possibility for the bees to forage for resources. The observations described in this study highlight the remarkable adaptations of *A. d. dorsata* and its co-evolved mites, and fundamentally challenges previous assumptions about the survival limitations of *A. d. dorsata* bees and *Tropilaelaps* mites. The interception of *A. d. dorsata* carrying *T. mercedesae* mites aboard a vessel inbound to a USA port highlights the importance of proactive surveillance and diagnostic readiness. Effective policies and mitigation measures prevented the entry of *T. mercedesae* and the mite *K. morsei* in the USA. However, the co-occurrence of BQCV, DWV-B and *P. larvae* pathogens further indicates the multifaceted risks posed by exotic bee introductions. Our findings underscore the value of early detection and interagency coordination for protecting USA apiculture and agriculture.

## Data Availability

The datasets presented in this study can be found in online repositories. The names of the repository/repositories and accession number(s) can be found in the article/**Supplementary Material**.
