# Supplementary material for: Interception of an Apis dorsata swarm with Tropilaelaps mercedesae and Kuzinia morsei mites on a cargo vessel inbound to the United States
Source: Front Insect Sci. 2026 May 8;6:1829350. doi: 10.3389/finsc.2026.1829350 (PMC13194098; doi:10.3389/finsc.2026.1829350)
Supplement: Supplementary file 1 [file Table1.docx]

**Supplemental Table S1:** Accessions, collection locations, and references for public data used in analyses. References marked as “Unpublished” indicate direct submissions to GenBank that are not associated with a publication. * indicates sequence was used for haplotype network

| **GenBank Accession** | **Species** | **Collection location** | **Reference** |
| --- | --- | --- | --- |
| EF025423.1* | *Tropilaelaps mercedesae* | Indonesia | Anderson and Morgan 2007 |
| EF025424.1* | *Tropilaelaps mercedesae* | Indonesia | Anderson and Morgan 2007 |
| EF025425.1* | *Tropilaelaps mercedesae* | Borneo | Anderson and Morgan 2007 |
| EF025426.1* | *Tropilaelaps mercedesae* | Borneo | Anderson and Morgan 2007 |
| EF025427.1* | *Tropilaelaps mercedesae* | Borneo | Anderson and Morgan 2007 |
| EF025428.1* | *Tropilaelaps mercedesae* | Borneo | Anderson and Morgan 2007 |
| EF025429.1* | *Tropilaelaps mercedesae* | China | Anderson and Morgan 2007 |
| EF025430.1* | *Tropilaelaps mercedesae* | China | Anderson and Morgan 2007 |
| EF025431.1* | *Tropilaelaps mercedesae* | China | Anderson and Morgan 2007 |
| EF025432.1* | *Tropilaelaps mercedesae* | China | Anderson and Morgan 2007 |
| EF025433.1* | *Tropilaelaps mercedesae* | India | Anderson and Morgan 2007 |
| EF025434.1* | *Tropilaelaps mercedesae* | Indonesia | Anderson and Morgan 2007 |
| EF025435.1* | *Tropilaelaps mercedesae* | Indonesia | Anderson and Morgan 2007 |
| EF025436.1* | *Tropilaelaps mercedesae* | Indonesia | Anderson and Morgan 2007 |
| EF025437.1* | *Tropilaelaps mercedesae* | Mainland Asia | Anderson and Morgan 2007 |
| EF025438.1* | *Tropilaelaps mercedesae* | Malaysia | Anderson and Morgan 2007 |
| EF025439.1* | *Tropilaelaps mercedesae* | Malaysia | Anderson and Morgan 2007 |
| EF025440.1* | *Tropilaelaps mercedesae* | Philippines | Anderson and Morgan 2007 |
| EF025441.1* | *Tropilaelaps mercedesae* | Sri Lanka | Anderson and Morgan 2007 |
| EF025442.1* | *Tropilaelaps mercedesae* | Sri Lanka | Anderson and Morgan 2007 |
| EF025443.1* | *Tropilaelaps mercedesae* | Indonesia | Anderson and Morgan 2007 |
| EF025444.1* | *Tropilaelaps mercedesae* | Indonesia | Anderson and Morgan 2007 |
| EF025445.1* | *Tropilaelaps mercedesae* | Thailand | Anderson and Morgan 2007 |
| EF025446.1* | *Tropilaelaps mercedesae* | Thailand | Anderson and Morgan 2007 |
| EF025447.1* | *Tropilaelaps mercedesae* | Vietnam | Anderson and Morgan 2007 |
| EF025448.1* | *Tropilaelaps mercedesae* | Vietnam | Anderson and Morgan 2007 |
| KY865176.1 | *Varroa destructor* | Thailand | Pakwan et al. 2017 |
| KY865177.1 | *Varroa destructor* | Thailand | Pakwan et al. 2017 |
| KY865178.1 | *Varroa destructor* | Thailand | Pakwan et al. 2017 |
| LC474394.1* | *Tropilaelaps mercedesae* | Papua New Guinea | Del Cont et al. 2021 |
| LC474395.1* | *Tropilaelaps mercedesae* | Papua New Guinea | Del Cont et al. 2021 |
| LC474396.1* | *Tropilaelaps mercedesae* | Indonesia | Del Cont et al. 2021 |
| LC474397.1* | *Tropilaelaps mercedesae* | Indonesia | Del Cont et al. 2021 |
| LC474398.1* | *Tropilaelaps mercedesae* | Indonesia | Del Cont et al. 2021 |
| LC474399.1* | *Tropilaelaps mercedesae* | Indonesia | Del Cont et al. 2021 |
| LC474400.1* | *Tropilaelaps mercedesae* | Sri Lanka | Del Cont et al. 2021 |
| LC474401.1* | *Tropilaelaps mercedesae* | Vietnam | Del Cont et al. 2021 |
| LC474405.1* | *Tropilaelaps mercedesae* | Pakistan | Del Cont et al. 2021 |
| MW337212.1 | *Tropilaelaps mercedesae* | India | Unpublished |
| MW337213.1 | *Tropilaelaps mercedesae* | India | Unpublished |
| MW337214.1 | *Tropilaelaps mercedesae* | India | Unpublished |
| MW487862.1 | *Tropilaelaps mercedesae* | India | Unpublished |
| MW487863.1 | *Tropilaelaps mercedesae* | India | Unpublished |
| MW487864.1 | *Tropilaelaps mercedesae* | India | Unpublished |
| MW504321.1 | *Tropilaelaps mercedesae* | Thailand | Unpublished |
| MW504322.1 | *Tropilaelaps mercedesae* | Thailand | Unpublished |
| MW504323.1 | *Tropilaelaps mercedesae* | Thailand | Unpublished |
| MW504324.1 | *Tropilaelaps mercedesae* | Thailand | Unpublished |
| MW504325.1 | *Tropilaelaps mercedesae* | Thailand | Unpublished |
| MW504326.1 | *Tropilaelaps mercedesae* | Thailand | Unpublished |
| MW504327.1 | *Tropilaelaps mercedesae* | Thailand | Unpublished |
| MW504328.1 | *Tropilaelaps mercedesae* | Thailand | Unpublished |
| MW504329.1 | *Tropilaelaps mercedesae* | Thailand | Unpublished |
| MW504330.1 | *Tropilaelaps mercedesae* | Thailand | Unpublished |
| MW504331.1 | *Tropilaelaps mercedesae* | Thailand | Unpublished |
| MW504332.1 | *Tropilaelaps mercedesae* | Thailand | Unpublished |
| MW535682.1 | *Tropilaelaps mercedesae* | Thailand | Unpublished |
| MW535683.1 | *Tropilaelaps mercedesae* | Thailand | Unpublished |
| MW535684.1 | *Tropilaelaps mercedesae* | Thailand | Unpublished |
| MW535685.1 | *Tropilaelaps mercedesae* | Thailand | Unpublished |
| MW535686.1 | *Tropilaelaps mercedesae* | Thailand | Unpublished |
| MW535687.1 | *Tropilaelaps mercedesae* | Thailand | Unpublished |
| MW535688.1 | *Tropilaelaps mercedesae* | Thailand | Unpublished |
| MW535689.1 | *Tropilaelaps mercedesae* | Thailand | Unpublished |
| MW535690.1 | *Tropilaelaps mercedesae* | Thailand | Unpublished |
| MW535691.1 | *Tropilaelaps mercedesae* | Thailand | Unpublished |
| MW535692.1 | *Tropilaelaps mercedesae* | Thailand | Unpublished |
| MW535693.1 | *Tropilaelaps mercedesae* | Thailand | Unpublished |
| MW725323.1 | *Tropilaelaps mercedesae* | South Korea | Truong et al. 2023 |
| MW725324.1 | *Tropilaelaps mercedesae* | South Korea | Truong et al. 2023 |
| MW725325.1 | *Tropilaelaps mercedesae* | South Korea | Truong et al. 2023 |
| MW725326.1 | *Tropilaelaps mercedesae* | South Korea | Truong et al. 2023 |
| MW725327.1 | *Tropilaelaps mercedesae* | South Korea | Truong et al. 2023 |
| MW725328.1 | *Tropilaelaps mercedesae* | South Korea | Truong et al. 2023 |
| MW725329.1 | *Tropilaelaps mercedesae* | South Korea | Truong et al. 2023 |
| MW725330.1 | *Tropilaelaps mercedesae* | South Korea | Truong et al. 2023 |
| MW725331.1 | *Tropilaelaps mercedesae* | South Korea | Truong et al. 2023 |
| MW725332.1 | *Tropilaelaps mercedesae* | South Korea | Truong et al. 2023 |
| MW725333.1 | *Tropilaelaps mercedesae* | South Korea | Truong et al. 2023 |
| OK157434.1 | *Tropilaelaps mercedesae* | India | Unpublished |
| OK188792.1 | *Tropilaelaps mercedesae* | India | Unpublished |
| OR165740.1* | *Tropilaelaps mercedesae* | Nepal | Namin et al. 2024 |
| OR165741.1* | *Tropilaelaps mercedesae* | Nepal | Namin et al. 2024 |
| OR165742.1* | *Tropilaelaps mercedesae* | Nepal | Namin et al. 2024 |
| OR165743.1* | *Tropilaelaps mercedesae* | Nepal | Namin et al. 2024 |
| OR165744.1* | *Tropilaelaps mercedesae* | Nepal | Namin et al. 2024 |
| OR165745.1* | *Tropilaelaps mercedesae* | South Korea | Namin et al. 2024 |
| OR165746.1* | *Tropilaelaps mercedesae* | South Korea | Namin et al. 2024 |
| OR165747.1* | *Tropilaelaps mercedesae* | South Korea | Namin et al. 2024 |
| OR165748.1* | *Tropilaelaps mercedesae* | South Korea | Namin et al. 2024 |
| OR165749.1* | *Tropilaelaps mercedesae* | South Korea | Namin et al. 2024 |
| OR165750.1* | *Tropilaelaps mercedesae* | South Korea | Namin et al. 2024 |
| OR165751.1* | *Tropilaelaps mercedesae* | South Korea | Namin et al. 2024 |
| OR165752.1* | *Tropilaelaps mercedesae* | South Korea | Namin et al. 2024 |
| OR165753.1* | *Tropilaelaps mercedesae* | South Korea | Namin et al. 2024 |
| OR165754.1* | *Tropilaelaps mercedesae* | South Korea | Namin et al. 2024 |
| OR165755.1* | *Tropilaelaps mercedesae* | South Korea | Namin et al. 2024 |
| OR165756.1* | *Tropilaelaps mercedesae* | South Korea | Namin et al. 2024 |
| OR165757.1* | *Tropilaelaps mercedesae* | South Korea | Namin et al. 2024 |
| OR165758.1* | *Tropilaelaps mercedesae* | South Korea | Namin et al. 2024 |
| OR165759.1* | *Tropilaelaps mercedesae* | South Korea | Namin et al. 2024 |
| OR165760.1* | *Tropilaelaps mercedesae* | South Korea | Namin et al. 2024 |
| OR165761.1* | *Tropilaelaps mercedesae* | South Korea | Namin et al. 2024 |
| OR165762.1* | *Tropilaelaps mercedesae* | South Korea | Namin et al. 2024 |
| OR165763.1* | *Tropilaelaps mercedesae* | South Korea | Namin et al. 2024 |
| OR165764.1* | *Tropilaelaps mercedesae* | South Korea | Namin et al. 2024 |
| OR165765.1* | *Tropilaelaps mercedesae* | South Korea | Namin et al. 2024 |
| OR165766.1* | *Tropilaelaps mercedesae* | South Korea | Namin et al. 2024 |
| OR165767.1* | *Tropilaelaps mercedesae* | South Korea | Namin et al. 2024 |
| OR165768.1* | *Tropilaelaps mercedesae* | South Korea | Namin et al. 2024 |
| OR165769.1* | *Tropilaelaps mercedesae* | South Korea | Namin et al. 2024 |
| OR165770.1* | *Tropilaelaps mercedesae* | South Korea | Namin et al. 2024 |
| OR165771.1* | *Tropilaelaps mercedesae* | South Korea | Namin et al. 2024 |
| OR165772.1* | *Tropilaelaps mercedesae* | South Korea | Namin et al. 2024 |
| OR165773.1* | *Tropilaelaps mercedesae* | South Korea | Namin et al. 2024 |
| OR165774.1* | *Tropilaelaps mercedesae* | South Korea | Namin et al. 2024 |
| OR165775.1* | *Tropilaelaps mercedesae* | South Korea | Namin et al. 2024 |
| OR165776.1* | *Tropilaelaps mercedesae* | South Korea | Namin et al. 2024 |
| OR165777.1* | *Tropilaelaps mercedesae* | South Korea | Namin et al. 2024 |
| OR165778.1* | *Tropilaelaps mercedesae* | South Korea | Namin et al. 2024 |
| OR165779.1* | *Tropilaelaps mercedesae* | South Korea | Namin et al. 2024 |
| OR165780.1* | *Tropilaelaps mercedesae* | South Korea | Namin et al. 2024 |
| OR165781.1* | *Tropilaelaps mercedesae* | South Korea | Namin et al. 2024 |
| OR165782.1* | *Tropilaelaps mercedesae* | South Korea | Namin et al. 2024 |
| OR165783.1* | *Tropilaelaps mercedesae* | South Korea | Namin et al. 2024 |
| OR165784.1* | *Tropilaelaps mercedesae* | South Korea | Namin et al. 2024 |
| OR165785.1* | *Tropilaelaps mercedesae* | Uzbekistan | Namin et al. 2024 |
| OR165786.1* | *Tropilaelaps mercedesae* | Uzbekistan | Namin et al. 2024 |
| OR165787.1* | *Tropilaelaps mercedesae* | Uzbekistan | Namin et al. 2024 |
| OR400173.1 | *Tropilaelaps mercedesae* | China | BioProject PRJNA343868 |
| OR488780.1 | *Tropilaelaps mercedesae* | India | Unpublished |
| OR965215.1 | *Tropilaelaps mercedesae* | Russia | Brandorf et al. 2025 |
| PQ049741.1 | *Tropilaelaps mercedesae* | Georgia | Janashia et al. 2024 |
| PQ455194.1 | *Tropilaelaps mercedesae* | Uzbekistan | Unpublished |
| PX136173.1 | *Tropilaelaps mercedesae* | Uzbekistan | Unpublished |
| PX136174.1 | *Tropilaelaps mercedesae* | Uzbekistan | Unpublished |
| PX136176.1 | *Tropilaelaps mercedesae* | Uzbekistan | Unpublished |
| PX136177.1 | *Tropilaelaps mercedesae* | Uzbekistan | Unpublished |
| PX136552.1 | *Tropilaelaps mercedesae* | Uzbekistan | Unpublished |
| PX136553.1 | *Tropilaelaps mercedesae* | Uzbekistan | Unpublished |
| PX136554.1 | *Tropilaelaps mercedesae* | Uzbekistan | Unpublished |
| MT670341.1 | *Apis dorsata dorsata* | Thailand | Unpublished |
| MT670342.1 | *Apis dorsata dorsata* | Thailand | Unpublished |
| MT670343.1 | *Apis dorsata dorsata* | Thailand | Unpublished |
| MT670344.1 | *Apis dorsata dorsata* | Thailand | Unpublished |
| MT679368.1 | *Apis dorsata dorsata* | Vietnam | Unpublished |
| MT679369.1 | *Apis dorsata dorsata* | Vietnam | Unpublished |
| MT679370.1 | *Apis dorsata dorsata* | Vietnam | Unpublished |
| MT679371.1 | *Apis dorsata dorsata* | Vietnam | Unpublished |
| MT679372.1 | *Apis dorsata dorsata* | Vietnam | Unpublished |
| MT679373.1 | *Apis dorsata dorsata* | Vietnam | Unpublished |
| MT679383.1 | *Apis dorsata dorsata* | Myanmar | Unpublished |
| MT679384.1 | *Apis dorsata dorsata* | Myanmar | Unpublished |
| MT679385.1 | *Apis dorsata dorsata* | Myanmar | Unpublished |
| MT679386.1 | *Apis dorsata dorsata* | Myanmar | Unpublished |
| MT679387.1 | *Apis dorsata dorsata* | Myanmar | Unpublished |
| MT679388.1 | *Apis dorsata dorsata* | Myanmar | Unpublished |
| MT679420.1 | *Apis dorsata dorsata* | Vietnam | Unpublished |
| MW504351.1 | *Apis dorsata dorsata* | Thailand | Unpublished |
| MW504352.1 | *Apis dorsata dorsata* | Thailand | Unpublished |
| MW504353.1 | *Apis dorsata dorsata* | Thailand | Unpublished |
| MW600451.1 | *Apis dorsata dorsata* | India | Unpublished |
| MW600452.1 | *Apis dorsata dorsata* | India | Unpublished |
| MZ420494.1 | *Apis dorsata dorsata* | India | Unpublished |
| MZ420495.1 | *Apis dorsata dorsata* | India | Unpublished |
| OM766176.1 | *Apis dorsata dorsata* | India | Unpublished |
| ON209533.1 | *Apis dorsata dorsata* | India | Unpublished |
| ON259323.1 | *Apis dorsata dorsata* | India | Unpublished |
| ON259531.1 | *Apis dorsata dorsata* | India | Unpublished |
| ON506691.1 | *Apis dorsata dorsata* | India | Bhat et al. 2022 |
| OP132602.1 | *Apis dorsata dorsata* | Cambodia | Unpublished |
| OP132603.1 | *Apis dorsata dorsata* | Cambodia | Unpublished |
| OP132604.1 | *Apis dorsata dorsata* | Cambodia | Unpublished |
| OP435372.1 | *Apis dorsata dorsata* | Bangladesh | Unpublished |
| OP720963.1 | *Apis dorsata dorsata* | Laos | Unpublished |
| OQ346356.1 | *Apis dorsata dorsata* | India | Unpublished |
| OQ346372.1 | *Apis dorsata dorsata* | India | Unpublished |
| OQ346374.1 | *Apis dorsata dorsata* | India | Unpublished |
| OQ346375.1 | *Apis dorsata dorsata* | India | Unpublished |
| OQ346377.1 | *Apis dorsata dorsata* | India | Unpublished |
| OQ439640.1 | *Apis dorsata dorsata** | India | Unpublished |
| OQ439641.1 | *Apis dorsata dorsata* | India | Unpublished |
| OQ699254.1 | *Apis dorsata dorsata* | Thailand | Unpublished |
| OQ699255.1 | *Apis dorsata dorsata* | Thailand | Unpublished |
| OR235194.1 | *Apis dorsata dorsata* | Thailand | Voraphab et al 2024 |
| OR235195.1 | *Apis dorsata dorsata* | Thailand | Voraphab et al 2024 |
| OR235196.1 | *Apis dorsata dorsata* | Thailand | Voraphab et al 2024 |
| OR235197.1 | *Apis dorsata dorsata* | Thailand | Voraphab et al 2024 |
| OR235198.1 | *Apis dorsata dorsata* | Thailand | Voraphab et al 2024 |
| OR235199.1 | *Apis dorsata dorsata* | Thailand | Voraphab et al 2024 |
| OR235200.1 | *Apis dorsata dorsata* | Thailand | Voraphab et al 2024 |
| OR235201.1 | *Apis dorsata dorsata* | Thailand | Voraphab et al 2024 |
| OR235202.1 | *Apis dorsata dorsata* | Thailand | Voraphab et al 2024 |
| OR235203.1 | *Apis dorsata dorsata* | Thailand | Voraphab et al 2024 |
| OR235204.1 | *Apis dorsata dorsata* | Thailand | Voraphab et al 2024 |
| OR235205.1 | *Apis dorsata dorsata* | Thailand | Voraphab et al 2024 |
| OR235206.1 | *Apis dorsata dorsata* | Thailand | Voraphab et al 2024 |
| OR235207.1 | *Apis dorsata dorsata* | Thailand | Voraphab et al 2024 |
| OR267460.1 | *Apis dorsata dorsata* | Thailand | Unpublished |
| OR267461.1 | *Apis dorsata dorsata* | Thailand | Unpublished |
| OR563663.1 | *Apis dorsata dorsata* | Malaysia | Unpublished |
| OR563665.1 | *Apis dorsata dorsata* | Malaysia | Unpublished |
| OR563673.1 | *Apis dorsata dorsata* | Malaysia | Unpublished |
| OR563674.1 | *Apis dorsata dorsata* | Malaysia | Unpublished |
| OR563675.1 | *Apis dorsata dorsata* | Malaysia | Unpublished |
| OR563676.1 | *Apis dorsata dorsata* | Malaysia | Unpublished |
| OR921350.1 | *Apis dorsata dorsata* | Bangladesh | Unpublished |
| PP101332.1 | *Apis dorsata dorsata** | India | Unpublished |
| PP388921.1 | *Apis florea* | United Arab Emirates | Unpublished |
| PP832980.1 | *Apis dorsata dorsata** | India | Bhatta et al. 2024 |
| PP832981.1 | *Apis dorsata dorsata** | India | Bhatta et al. 2024 |
| PP832982.1 | *Apis dorsata dorsata** | India | Bhatta et al. 2024 |
| PP832983.1 | *Apis dorsata dorsata** | India | Bhatta et al. 2024 |
| PP832984.1 | *Apis dorsata dorsata** | India | Bhatta et al. 2024 |
| PP832985.1 | *Apis dorsata dorsata* | Pakistan | Bhatta et al. 2024 |
| PP832986.1 | *Apis dorsata dorsata* | India | Bhatta et al. 2024 |
| PP832987.1 | *Apis dorsata dorsata* | India | Bhatta et al. 2024 |
| PP832988.1 | *Apis dorsata dorsata* | Nepal | Bhatta et al. 2024 |
| PP832989.1 | *Apis dorsata dorsata* | Nepal | Bhatta et al. 2024 |
| PP832990.1 | *Apis dorsata dorsata* | Nepal | Bhatta et al. 2024 |
| PP832991.1 | *Apis dorsata dorsata* | Nepal | Bhatta et al. 2024 |
| PP832992.1 | *Apis dorsata dorsata* | India | Bhatta et al. 2024 |
| PP832993.1 | *Apis dorsata dorsata* | Thailand | Bhatta et al. 2024 |
| PP832994.1 | *Apis dorsata dorsata* | Malaysia | Bhatta et al. 2024 |
| PP832995.1 | *Apis dorsata dorsata* | Malaysia | Bhatta et al. 2024 |
| PP832996.1 | *Apis dorsata dorsata* | Philippines | Bhatta et al. 2024 |
| PP832997.1 | *Apis dorsata dorsata* | Indonesia | Bhatta et al. 2024 |
| PP832998.1 | *Apis dorsata dorsata* | Indonesia | Bhatta et al. 2024 |
| PP832999.1 | *Apis dorsata dorsata* | Indonesia | Bhatta et al. 2024 |
| PP833000.1 | *Apis dorsata brevilingua* | Philippines | Bhatta et al. 2024 |
| PP833001.1 | *Apis dorsata brevilingua* | Philippines | Bhatta et al. 2024 |
| PP833002.1 | *Apis dorsata brevilingua* | Philippines | Bhatta et al. 2024 |
| PP833003.1 | *Apis dorsata binghami* | Indonesia | Bhatta et al. 2024 |
| PP833004.1 | *Apis dorsata binghami* | Indonesia | Bhatta et al. 2024 |
| PP833005.1 | *Apis dorsata binghami* | Indonesia | Bhatta et al. 2024 |
| PQ036158.1 | *Apis dorsata dorsata* | India | Unpublished |
| PQ407639.1 | *Apis dorsata dorsata* | Bangladesh | Unpublished |
| PQ409429.1 | *Apis dorsata dorsata* | Bangladesh | Unpublished |
| PQ481961.1 | *Apis dorsata dorsata* | Bangladesh | Unpublished |
| PQ835499.1 | *Apis dorsata dorsata** | India | Unpublished |
| PQ867463.1 | *Apis florea* | India | Unpublished |
| PV480534.1 | *Apis dorsata dorsata* | Myanmar | Bawm et al. 2025 |
| PV480535.1 | *Apis dorsata dorsata* | Myanmar | Bawm et al. 2025 |
| PV480536.1 | *Apis dorsata dorsata* | Myanmar | Bawm et al. 2025 |
| PV611191.1 | *Apis florea* | India | Unpublished |
| PV612240.1 | *Apis dorsata dorsata** | India | Unpublished |
